# Impact of Somatic and Addictive Comorbidities on the Quality of Life of Patients With Schizoaffective Disorder: A cross-sectional study

**DOI:** 10.1192/j.eurpsy.2023.333

**Published:** 2023-07-19

**Authors:** M. Asgharzadeh, W. Bouali, S. Khouadja, R. Ben Soussia, S. Younes, L. Zarrouk

**Affiliations:** Psychiatry Department, Taher Sfar University Hospital of Mahdia, Mahdia, Tunisia

## Abstract

**Introduction:**

Schizoaffective disorder is a mental health disorder frequently associated with somatic and addictive comorbidities. This association can be dangerous as it may change the expression of the disease and its prognosis and may even affect the quality of life (QOL) and the overall functioning of patients.

**Objectives:**

This study aimed to investigate the impact of somatic and addictive comorbidities on QOL and the overall functioning of patients with schizoaffective disorder.

**Methods:**

This paper is a cross-sectional descriptive study conducted at the *Department of Psychiatry within outpatient settings* over six months. We evaluated the QOL using the SF-36 and Global Assessment of Functioning scale (GAF). We included all patients suffering from a schizoaffective disorder and excluded patients with associated mental impairment, those we could not assess because of another disability, and those with missing records.

**Results:**

Fifty-two patients with schizoaffective disorder met our inclusion criteria with an average age of 38. This study found somatic comorbidities in 30.8% of patients, where diabetes ranked first (13.5%), followed by high blood pressure (9.6%). We noted addictive comorbidities in 63.5% of patients, with tobacco, alcohol, and Cannabis being the most consumed substances, with respective rates of 57.7 %,28.8 %, and 13.5%.

The QOL assessment revealed an impaired QOL score in 80.8% of patients, whereas 65.4% had a GAF score lower than 70 indicating a deterioration of functioning level. Our results showed that dimension D1 (physical activity) of the SF-36 was strongly influenced by somatic comorbidities, according to an analytical investigation of the association between these two variables (p = 10-4). We also found that the deterioration in the patient’s global functioning was not significantly related to somatic comorbidities (p = 0.28). Furthermore, our studies showed no association between impaired quality of life and substance abuse.

**Conclusions:**

Somatic comorbidities and substance abuse have a detrimental effect on patients with pre-existent schizoaffective disorder, and the goals of patient care with a schizoaffective illness go beyond the remission of clinical symptoms to the improvement of quality of life and socio-professional functioning.

**Disclosure of Interest:**

None Declared

